# Biological activities and phytochemical characterization of *Sideritis germanicopolitana* subsp. *viridis* and *S. libanotica* subsp. *linearis* extracts and extract-loaded nanoparticles

**DOI:** 10.3389/fphar.2025.1508762

**Published:** 2025-03-18

**Authors:** Turgut Taşkın, Beyza Nur Yılmaz, Shalaleh Hasan Niari Niar, Mizgin Ermanoğlu, Duygu Taşkın, İsmail Şenkardeş, Talip Şahin, Elif Çalışkan Salihi, Ali Demir Sezer, Oya Kerimoğlu, Hatice Kübra Elçioğlu

**Affiliations:** ^1^ Department of Pharmacognosy, Faculty of Pharmacy, Marmara University, Istanbul, Türkiye; ^2^ Marmara Pharmacy Drug and Innovative Product Development Unit, Faculty of Pharmacy, Marmara University, Istanbul, Türkiye; ^3^ Department of Pharnacognosy, Institute of Health Sciences, Marmara University Istanbul, Istanbul, Türkiye; ^4^ Department of Basic Pharmaceutical Sciences, Institute of Health Sciences, Marmara University Istanbul, Istanbul, Türkiye; ^5^ Department of Basic Pharmaceutical Sciences, Faculty of Pharmacy, Marmara University, Istanbul, Türkiye; ^6^ Department of Analytical Chemistry, Faculty of Pharmacy, University of Health Sciences, Istanbul, Türkiye; ^7^ Department of Pharmaceutical Botany, Faculty of Pharmacy, Marmara University, Istanbul, Türkiye; ^8^ Department of Biology, Institute of Science, Adıyaman University, Adıyaman, Türkiye; ^9^ Department of Pharmaceutical Biotechnology, Faculty of Pharmacy, Marmara University, Istanbul, Türkiye; ^10^ Department of Pharmaceutical Technology, Faculty of Pharmacy, Marmara University, Istanbul, Türkiye; ^11^ Department of Pharmacology, Faculty of Pharmacy, Marmara University, Istanbul, Türkiye

**Keywords:** nanoparticles, biological activity, medicinal plants, *Sideritis germanicopolitana* subsp. *viridis*, *S. libanotica* subsp. *linearis*, HPLC-DAD

## Abstract

**Introduction:**

The current study focuses on evaluating the biological activity and analysis of phytochemical content of extracts and extract-loaded nanoparticles from *Sideritis germanicopolitana* subsp. *viridis* (endemic, SGV) and *S. libanotica* subsp. *linearis* (SLL).

**Methods:**

Antioxidant activities of extracts and nanoparticles were investigated by DPPH, FRAP and CUPRAC methods. Enzyme inhibition potentials of extracts and nanoparticles were evaluated by Ellman and indophenol methods. Phytochemical contents were analyzed by HPLC-DAD. Plant extracts were encapsulated by the ionic gelation method which was modified in our laboratory using the green chemistry approach.

**Results and Discussion:**

It was found that the 70% ethanol extracts of SGV and SLL exhibited the highest antioxidant activity in terms of DPPH, FRAP and CUPRAC compared to other extracts. The findings showed that both 70% ethanol extract-loaded nanoparticles obtained from SGV and SLL showed lower DPPH radical scavenging, iron (III) reducing and copper (II) reducing activities compared to crude extracts. It was determined that the 70% extracts of SGV and SLL exhibited a higher potential to inhibit the enzyme urease than other extracts. The anti-urease activity of the nanoparticle loaded with SLL 70% ethanol extract was found to be greater than that of the nanoparticle made with SGV 70% ethanol extract. Furthermore, an analysis of the acetylcholinesterase enzyme inhibition capacity of various extracts from both plants revealed that the 70% ethanol extracts of each plant species had a greater potential for enzyme inhibition than the other extracts. The anticholinesterase activity of the nanoparticle loaded with SLL 70% extract was found to be higher than that of the nanoparticle loaded with SGV 70% ethanol extract. In this study the phenolic metabolites were examined, luteolin (27.44 μg/mg extract) and *p*-coumaric acid (20.03 μg/mg extract) were found at the highest concentration in the SGV plant while rosmarinic acid (8.70 μg/mg extract), caffeic acid (6.46 μg/mg extract) and *p*-coumaric acid (4.42 μg/mg extract) were found at the highest concentration in the SLL plant. However, the data demonstrated that the nanoparticles had lesser biological activity potential than crude extracts.

**Conclusion:**

The substantial biological activities of the nanoparticles developed as a result of this work showed that these formulations are suitable for use as antioxidant, anti-urease and anticholinesterase medicines in the future due to the benefits of using nanoparticles in the therapeutics such as the controlled release of the active agents and the diminished side effects.

## 1 Introduction

Reactive oxygen species (ROS) and reactive nitrogen species (RNS) have a role in the organism, both harmful and beneficial. In the body, there is a balance between free radicals and endogenous antioxidants, which are in homeostasis. When this balance is disturbed in favor of free radicals (ROS, RNS, etc.), oxidative stress occurs (Valko et al., 2007; Barja, 2004; Fang et al., 2002). As a result, it causes oxidative damage on DNA, RNA, lipids and proteins. This situation is the cause of many diseases such as cardiovascular diseases, ischemic injury, injuries, rheumatoid arthritis, diabetes, Alzheimer’s, Parkinson’s and especially cancer. In recent studies, oxidative stress has been found to exacerbate the course of coronavirus disease (COVID-19) and complicate the treatment. For this reason, the discovery of exogenous antioxidant agents with high therapeutic effects and low side effects remains important (Bakadia et al., 2021). *Helicobacter pylori* is a gram-negative microaerophilic bacterium that infects up to 50% of the world’s human population. The eradication of *H. pylori* is known to cause gastritis, peptic ulcers, gastric cancers. Through the enzyme urease, *H. pylori* breaks down urea into ammonia and carbon dioxide and colonizes the mucosa by freeing itself from the acidic pH of the gastric surface (Stingl and De Reuse, 2005; Azizian et al., 2012; Graham and Miftahussurur, 2018). Alzheimer’s disease (AD) is a cholinergic neurodegenerative disease that affects the brain which is irreversible. It is characterized by progressive loss of memory and general cognitive decline. The cholinergic system is being targeted in the development of anti-Alzheimer medications because it is crucial to the regulation of learning and memory processes. Through the inhibition of the enzyme acetylcholinesterase (AChE), which hydrolyzes acetylcholine, cholinesterase inhibitors directly increase cholinergic transmission. Additionally, it has been demonstrated that in the early phases of senile plaque formation, both butyrylcholinesterase (BuChE) and acetylcholinesterase are crucial for the aggregation of amyloid plaque (Fisher et al., 2012; Anand and Singh, 2013; Mishra et al., 2019).

Phytochemicals undergo digestion and degradation through the mouth, stomach, small and large intestines, then are absorbed from the digestive tract into the blood or lymphatic circulation and further distributed by diffusion or transport into the body circulation, followed by metabolization in body tissues by biochemical transformation or degradation, and final excretion through the renal, biliary or pulmonary pathways (Martinez-Perez et al., 2018; Holst and Williamson, 2008). The distribution and absorption of phytochemicals in the small intestine depend on their chemical structure and polarity. Nanoparticles used as carriers are designed to deliver phytochemicals to the target site with enhanced bioactivity. Since nanoparticles contain materials designed at the atomic or molecular level, they are generally small-sized nanostructures. Therefore, they can move more freely in the human body compared to larger materials. Nanotechnology plays a significant role in drug generation and controlled delivery to the target site and controlled release. Therefore, this technology provides numerous benefits in the treatment of chronic human diseases by site-specific and target-oriented delivery of drugs (Jahangirian et al., 2017; Martinez-Ballesta et al., 2018; Ahmad et al., 2021). The genus *Sideritis*, which is a member of Lamiaceae and has aromatic and medicinal properties. This genus is distributed in the Eastern and Western Mediterranean regions, as 46 species grow in the flora of Turkey and 31 of them are endemic (Tepe et al., 2006; Kan et al., 2018). Most of the *Sideritis* species grow in the high mountain regions and are called mountain tea by the locals. The infusion of the aerial part of *Sideritis* species is used in traditional folk medicine as a carminative, digestive aid, cough suppressant, diuretic (Formisano et al., 2015; Sevindik et al., 2021). *Sideritis* species has been the research subject because it has been used in different treatments for many years. Species belonging to this genus contain terpenes, flavonoids, essential oil, iridoids, coumarins and sterols, which provide this species with a wide range of bioactive effects, especially antimicrobial, antioxidant, antiinflammatory, antispasmodic, antiulcerative, anticonvulsant, carminative, analgesic and sedative effects (González-Burgos et al., 2011; Dincer et al., 2017). *Sideritis libanotica* subsp. *linearis* (Benth.) Bornm. is species growing in Turkey. The MeOH extract from the aerial parts of the plant was evaluated for its activity in the DPPH^•^, ABTS^•+^ and carotene-linoleic acid assays as well as for its total phenolic and flavonoid amounts (Tepe et al., 2006; Dincer et al., 2017; Atas et al., 2019). The perennial plant species *Sideritis germanicopolitana* subsp. *viridis* Hausskn. ex Bornm. is endemic to Turkey and occurs mainly in the Northern Anatolia. Its essential oil has been reported to contain metabolites such as α-pinene, myrcene, sabinene and β-pinene (Özkan et al., 2005; Bayan and Aksit, 2016). Only a limited number of studies on the biological activity and chemical content of both *Sideritis* species were found in the literature search (Tepe et al., 2006; Bayan and Aksit, 2016).

For the formulation of cosmetic, food, and pharmaceutical products which comprised of natural compounds showing biological activities; their stability and shelf life must be enhanced by protecting them from the environmental damage. Compounds such as polyphenols are not stable and make interactions easily, since they have unsaturated bonds in their molecular structure which makes them sensitive to the environmental factors such as oxidants, light exposure, heat, pH, water, and enzymatic activities. In order to protect the ingredients, encapsulation methods have been explored to improve the undesirable properties of the herbal extracts and also to enhance the stability and the delivery of them. The advantages of carrier systems are that, in addition to reducing toxicity, they provide controlled release and increase the bioavailability of the active ingredients (Hcini et al., 2021; Zeng et al., 2023). As a result, the objectives of this study are the preparation of *Sideritis germanicopolitana* subsp. *Viridis* and *Sideritis libanotica* subsp. *Linearis* extracts in order to investigate their *in vitro* biological activities; production of extract loaded nanoparticles; characterization of nanoparticles produced; quantitative analysis of phytochemical content of bioactive extracts and extracts-loaded nanoparticles.

## 2 Materials and methods

### 2.1 The preparation of plant extracts and the use of plant materials

*Sideritis germanicopolitana* subsp. *viridis* (SGV) and *Sideritis libanotica* subsp. *linearis* (SLL) species were identified by Assist. Prof. Dr. İsmail Şenkardeş. They were cataloged with the herbarium numbers 19155 and 22,881 and stored in the Faculty of Pharmacy’s Herbarium (MARE) at Marmara University. The plant samples were dried at the room temperature. The samples were then extracted by maceration with EtOH:distilled water (70:30) until colorless. Petroleum ether and chloroform extracts were from the crude 70% ethanol extract by liquid-liquid extraction method, respectively. A rotary vacuum evaporator was used to concentrate the six distinct plant extracts. Until examination, all of the extracted materials were kept at +4°C.

### 2.2 Preparation of nanoparticles loaded with plant extracts

Plant extracts were encapsulated by adopting ionic gelation method (Rajaonarivony et al., 1993) modified in our laboratory using the green chemistry approach. SGV encapsulated nanoparticles (SGV NPs) and SLL encapsulated nanoparticles (SLL NPs) were prepared using sodium alginate (SA) as a natural polymer in aqueous medium without using any additives or surface active agents. Firstly, 1% w/v aqueous solutions of SGV/SLL extracts were prepared and added dropwise into the 2% w/v aqueous solutions of SA in equal volumes under constant stirring. This mixture then added to a 3% w/v calcium chloride bath using the dripping technique. Gelation was observed while dripping and the formed gel was kept in dark medium at room temperature overnight. The next day, after washing with deionized water, the SGV NPs and the SLL NPs were left to dry for 48 h at room temperature and under ambient pressure (Çalışkan Salihi et al., 2025). After this slow drying process, they were dried at 45°C for 4 h and the dried nanoparticles were kept at airtight containers. Empty nanoparticles were also prepared using the same procedure for comparison purposes.

### 2.3 Characterization of nanoparticles loaded with plant extracts

Fourier-transform infrared (FTIR) spectroscopy was used to analyze the chemical functionalities in the structure of nanoparticle formulation. FTIR spectra of SGV NPs and SLL NPs were obtained from 4,000 to 500 cm^−1^ with an average resolution of 4 cm^−1^ (IRSpirit spectrometer, Shimadzu Corp, Kyoto, Japan). Surface morphology and shape of the nanoparticles were investigated by using Scanning electron microscopy (SEM). The powder samples were mounted on aluminum stubs and the nanoparticles were recorded by using the FEI Quanta 650 FEG SEM device. Sizes of the nanoparticles were measured by using the Zetasizer (Malvern Nano ZS) device applying dynamic light scattering (DLS) technique. Average diameter (Z-average) and the polydispersity index (PDI) of the nanoparticles were measured and compared. The encapsulation efficiency (EE, %) and the Loading capacity (LC, %) were determined by analyzing the filtrate using the UV-Visible Spectrophotometry (Shimadzu 2100S) using the predetermined calibration curves. The EE and the LC were then calculated using the Equations 1, 2, which are given below.

$$\text{EE }\left(\%\right)=\left(\text{Weight of the extract in the NPs}/|\text{Weight of the extract used}\right)\times 100$$ (1)

$$\text{LC }\left(\%\right)=\left(\text{Weight of the extract in the NPs}/|\text{Weight of the NPs }\right)\times 100$$ (2)

*In vitro* release of SGV and SLL from the nanoparticle formulations were studied spectrophotometrically (Shimadzu 2100S) using dialysis bags in PBS (phosphate-buffered saline, pH 7.4) medium. 25 mg of each nanoformulations and 50 mL of PBS was used for the release experiments. Release experiments were conducted in a thermostatic shaking water bath at 37°C. Samples were taken at predetermined time intervals (at 1 h, 2 h, 3 h, 4 h, 5 h, and 6 h) and the concentration of the samples were calculated by spectrophotometric method using the calibration curves prepared initially (Hashem et al., 2022).

### 2.4 Antioxidant activity

#### 2.4.1 FRAP assays

The FRAP reagent was stored at 37°C for 30 min. It consisted of 25 mL of 300 mM acetate buffer (pH 3.6), 2.5 mL of TPTZ solution, and 2.5 mL of 20 mM FeCl_3_. 10 μL of extracts/nanoparticles were combined with 190 µL of FRAP reagent, and after 4 min, the mixture’s absorbance at 593 nm was measured. The extracts’ FRAP values were presented as mM Fe^2+^/mg extract (Benzie and Strain, 1996).

#### 2.4.2 DPPH assays

240 µL of DPPH solution (0.1 mM) were added to the 10 µL of extracts/nanoparticles that had been obtained at various doses (0.5–3 mg/mL). Before being incubated for 30 min at 25°C, the produced mixtures were stirred for 1 min. Every day at 517 nm, the mixes’ absorbance values were measured. Under identical circumstances, the absorbance of the control sample was measured using 10 µL of methanol rather than the extract. The information gathered throughout the investigation is provided as IC_50_ = mg/mL (Wei et al., 2010).

#### 2.4.3 CUPRAC assays

The ability of extracts and nanoparticles to reduce copper (II) ions was evaluated using a method established by Apak et al., in 2004. In a nutshell, 60 µL of CuCl_2_·2H_2_O, 60 µL of neocuproine, and 60 µL of 1 M NH_4_Ac were mixed, then 60 µL of the extracts/nanoparticles were added, and finally 10 µL of ethanol was added to the mixture. The mixes’ absorbance was spectrophotometrically evaluated at 450 nm after 60 min against a reference solution that was made by substituting ethanol for the plant extracts. The extracts’/nanoparticles’ CUPRAC values were provided as mg trolox equivalent/mg extract (Apak et al., 2004).

#### 2.4.4 Total phenolic contents (FCR assay)

25 µL of extracts, 100 µL of Folin-Ciocalteu reagent (diluted 1/3 with distilled water) and 75 µL of 2% sodium carbonate solution were added to the plate. Following this, the mixture was left at room temperature for 2 hours and then the absorbance at 750 nm was measured by comparison with the reference. The total phenolic content of the extracts was expressed as mg gallic acid equivalents (GAE)/mg extract (Gülsoy Toplan, et al., 2022).

### 2.5 Enzyme assays

#### 2.5.1 Anti-urease activity

An enzyme solution (500 µL) and plant extracts/nanoparticles (100 µL) were combined and incubated for 30 min at 37°C. After adding 1,100 µL of urea to the mixture, it was left in an incubator set to 37°C for half an hour. After being taken out of the incubator, the mixture was combined with reagents R_1_ (1% phenol, 0.005% sodium nitroprusside) and R_2_ (0.5% NaOH, 0.1% sodium hypochlorite), and it was then incubated at 37°C for 2 hours. The absorbance of the mixture (635 nm) was measured in relation to a reference solution that was made by substituting a buffer solution for the urease enzyme solution (Ghous et al., 2010).

#### 2.5.2 Anticholinesterase activity

Using a microplate reader, the inhibition activities of acetylcholinesterase (AchE) were determined. Acetylthiocholine iodide was utilized as a substrate for the enzyme acetylcholinesterase, which is sourced from electrophorus electricus. The compound used to measure the activity was 5,5-dithiobis-2-nitrobenzoic acid (DTNB), which has a yellow color. Galantamine, an alkaloid-type medication that was extracted from the *Galanthus* plant, was utilized as a control. In summary, 40 µL of phosphate buffer solution (pH 8.2 0.1 M) was mixed with 20 µL of AchE and various extract/nanoparticles concentrations. For 10 minutes, this mixture was incubated at 25°C. Following incubation, the mixture was supplemented with 20 μL of AcI substrate and 100 μL of DTNB. The galantamine that was used as a standard underwent the same process. At 412 nm, 5-thio-2nitrobenzoic acid was measured with spectrophotometry. The following formula was used to determine the extracts’/nanoparticles anticholinesterase activity as a percentage of inhibition compared to the control (Ellman et al., 1961). The following formula was used to get the percentage of acetylcholinesterase inhibition: %I = (A_control_–A_sample_/A_control_)x100.

### 2.6 HPLC analysis of phytochemical metabolites

High pressure liquid chromatography (HPLC-DAD) system was used to determine the amount of phenolic metabolites contained in the 70% ethanol extracts from plants (Agilent Technologies 1260 Infinity, California, United States). Waters Nova-Pak C18 column (4 μm; 3.9 × 150 mm) was used to separate the metabolites in the plants. The metabolites were analyzed in the HPLC-DAD system using the following conditions: Mobile phase (A) consisted of water and 0.05% formic acid; mobile phase (B) consisted of acetonitrile and 0.05% formic acid. The following gradient program was applied: 0 min 5% B; 1 min 5% B; 20 min 30% B; 25 min 60% B; 28 min 60% B; 33 min 95% B; 35 min 95% B; 40 min 5% B. 20 μL of sample was injected into the system and the flow rate was 0.5 mL/min. The obtained 70% ethanol extracts were dissolved in methanol solvent and injected into the HPLC system after being filtered through a 0.45 μm syringe tip microfilter (Taşkın et al., 2021).

### 2.7 Statistical analysis

The Graphpad Prism 5 program was used to assess the study’s data. For p values less than 0.05, statistical differences between research groups were examined using ANOVA and Tukey’s multiple comparison test.

## 3 Results

### 3.1 The total phenolic contents

The total phenolic contents of many plant extracts were computed in this investigation. The 70% ethanol extracts from SLL (7.300 mg GAE/g extract) and SGV (7.122 mg GAE/g extract) had the highest concentration of phenolic metabolites. It is commonly recognized that there is frequently a linear relationship between phenolic chemicals and antioxidant activity. This study indicated that 70% ethanol extracts with abundant phenolic metabolites had better antioxidant activity than other extracts, which was in line with the literature. Furthermore, the total phenolic contents of the nanoparticles loaded with 70% ethanol extract were measured and compared to crude extracts in this investigation. The results indicated that the phenolic content of crude extracts was higher than that of nanoparticles. It was determined that the nanoparticles prepared from the SLL species (1.800 mg GAE/g extract) had a higher TPC value than the SGV nanoparticles (0.460 mg GAE/g extract) (Table 1).

**TABLE 1:** Antioxidant activity and total phenolic content of different extracts and extract-loaded nanoparticles.

Assays					Extracts						Standards
			SGV					SLL			
	Petroleum Ether	Chloroform	70% ethanol	70% ethanol loaded nanoparticle	Extract unloaded nanoparticle	Petroleum ether	Chloroform	70% ethanol	70% ethanol loaded nanoparticle	Extract unloadedna noparticle	Ascorbic acid
DPPH (IC_50_: mg/mL)	0.149 ± 0.010*	0.056 ± 0.006*	0.028 ± 0.002*	4.421 ± 1.079*	NA	1.891 ± 1.462*	0.041 ± 0.004*	0.039 ± 0.001*	3.154 ± 0.273*	NA	0.004 ± 0.001
FRAP (mMFeSO_4_/m g extract)	0.161 ± 0.005*	0.941 ± 0.051*	1.718 ± 0.094*	0.013 ± 0.001*	NA	0.368 ± 0.045*	1.297 ± 0.152*	1.448 ± 0.015*	0.018 ± 0.002*	NA	7.808 ± 0.353
CUPRAC (mMTE/mg extract)	0.194 ± 0.032*	1.306 ± 0.238*	2.538 ± 0.011*	0.022 ± 0.012*	NA	0.481 ± 0.030*	2.128 ± 0.180*	2.278 ± 0.182*	0.030 ± 0.028*	NA	5.831 ± 0.068
Total phenolicconte nt (mgGAE/g extract)	0.016 ± 0.017	2.759 ± 0.047	7.122 ± 0.588	0.460 ± 0.026	NA	3.325 ± 0.722	3.797 ± 0.379	7.300 ± 0.322	1.800 ± 0.164	NA	

Ascorbic acid positive control for DPPH, FRAP and CUPRAC, assays; DPPH, 2,2-diphenyl-1-picrylhydrazyl; CUPRAC, cupric ion reducing/antioxidant power; FRAP, ferric reducing antioxidant power; Values are mean of triplicate determination (n = 3) ± standard deviation; *P < 0.05 compared with the positive control; SGV: *S. germanicopolitana* subsp. *viridis;* SLL*: S. libanotica* subsp. *linearis*.

### 3.2 Phenolic metabolites analysis

**FIGURE 1:**
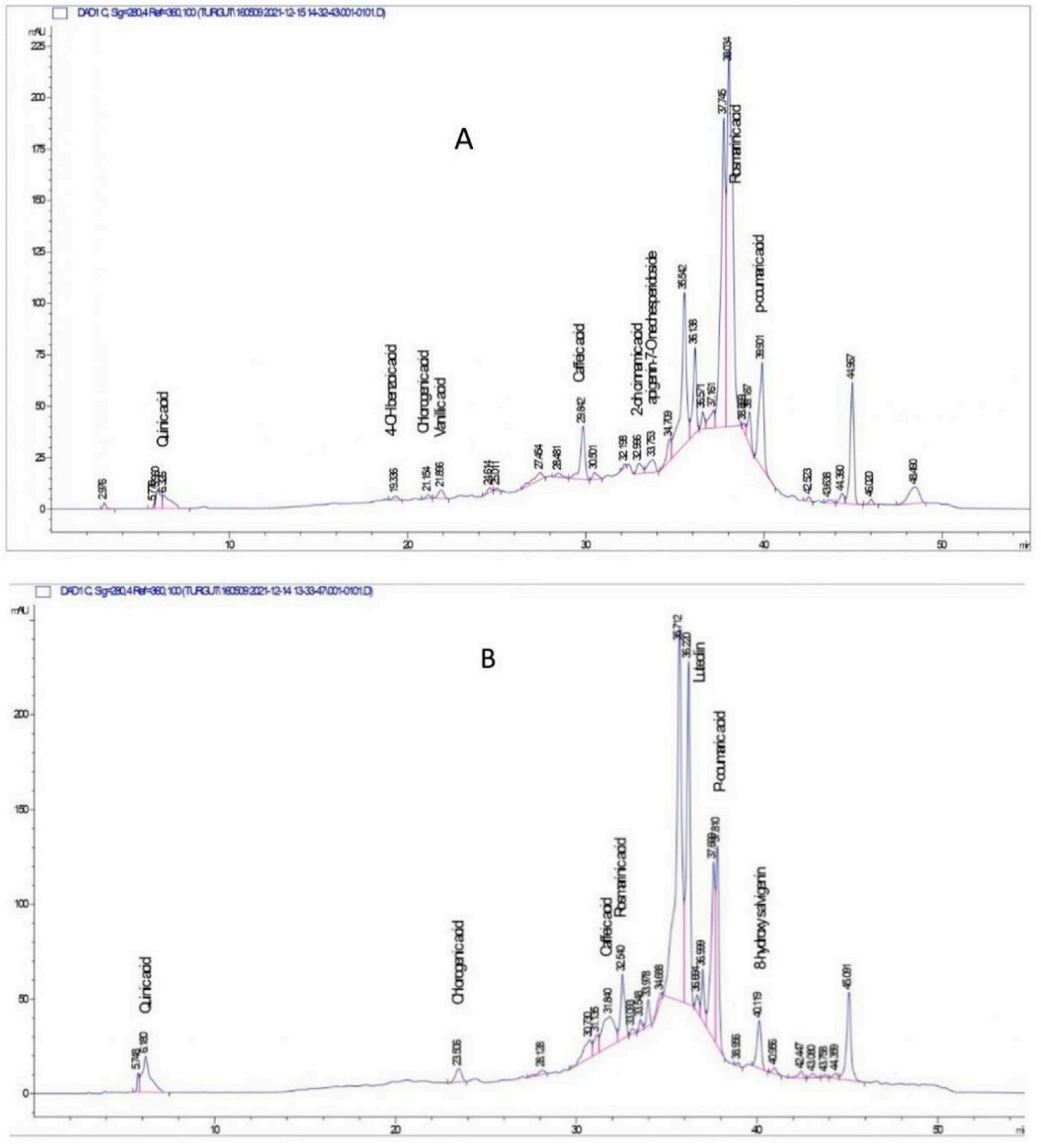
HPLC-DAD chromatogram of phenolic and flavonoid compounds detected from *Sideritis* species extracts. **(A)** *S. libanotica* subsp. *linearis* extract; **(B)** *S. germanicopolitana* subsp.*viridis* extract.

**FIGURE 2:**
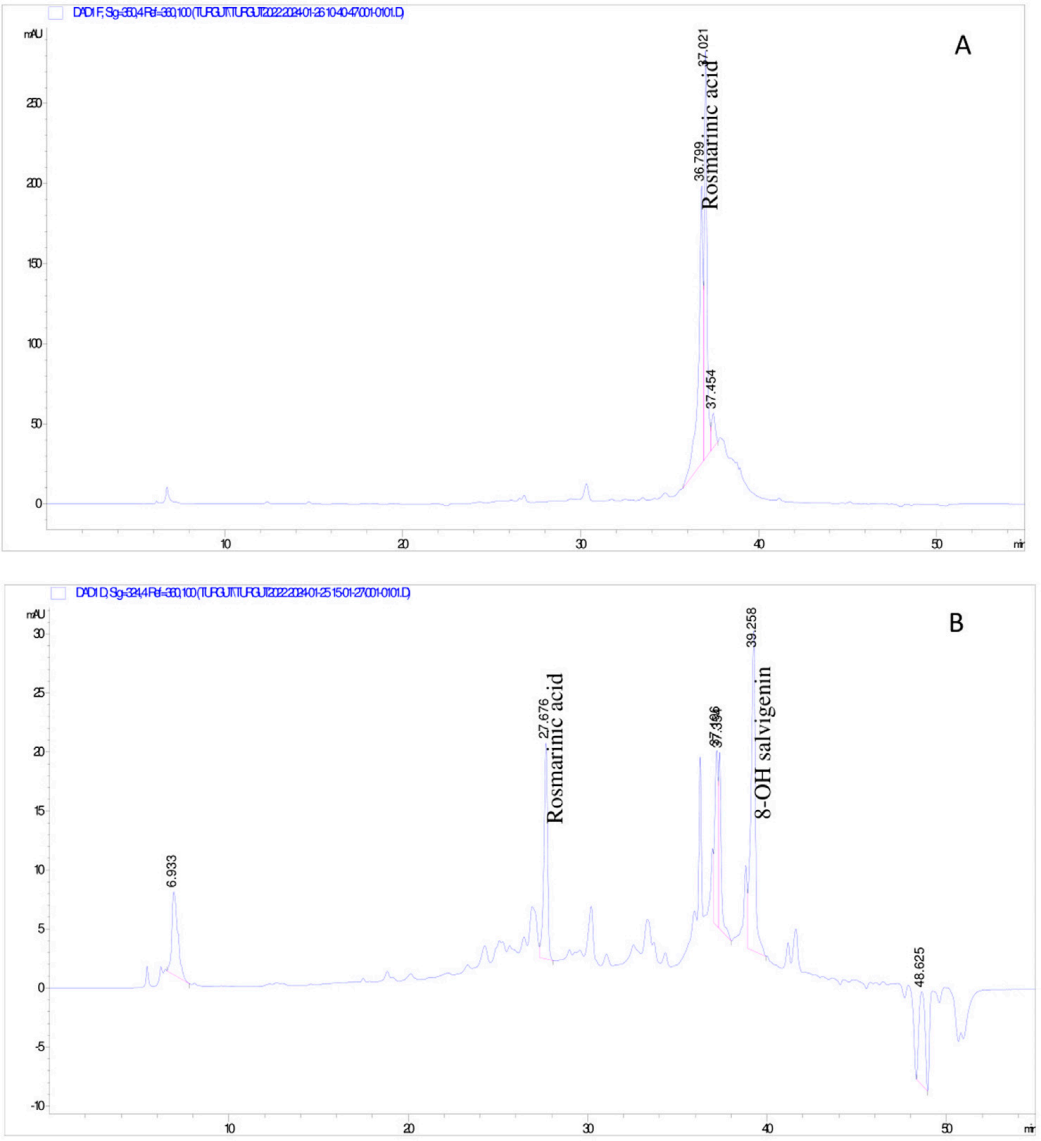
HPLC-DAD chromatogram of metabolites detected in nanoparticles. **(A)** *S. libanotica* subsp. *linearis* extract; **(B)** *S. germanicopolitana* subsp.*viridis* extract.

The composition of SGV and SLL extracts was examined both qualitatively and quantitatively since 70% ethanol extracts from plants shown considerable biological activity when compared to other extracts. Quinic acid, 4-hydroxybenzoic acid, chlorogenic acid, vanillic acid, caffeic acid, 2hydroxycinnamic acid, apigenin-7-*O*-neohesperidosid, rosmarinic acid and *p*-coumaric acid were found in the SLL 70% ethanol extract. The highest amounts of rosmarinic acid (8.70 µg analyte/mg extract), caffeic acid (6.46 µg analyte/mg extract) and *p*-coumaric acid (4.42 µg analyte/mg extracts) were determined in the extract of this plants (Table 2; Figure 1). Quinic acid, chlorogenic acid, caffeic acid, rosmarinic acid, luteolin, *p*-coumaric acid and 8-hydroxy salvigenin were detected in the SGV 70% ethanol extract. The major metabolites in this species were determined to be luteolin (27.44 µg analyte/mg extract) and *p*-coumaric acid (20.03 µg analyte/mg extract) (Table 3; Figure 1). In this investigation, chemotype differences between *Sideritis* species were found. 4-hydroxybenzoic acid, vanillic acid, 2- hydroxycinnamic acids, apigenin-7-*O*-neohesperidosid were observed only in the SLL species, while luteolin and 8-hydroxy salvigenin were analyzed in the SGV species. HPLC-DAD findings showed that both SLL (7.46 µg analyte/mg extract) and SGV (1.79 µg analyte/mg extract) nanoparticles contained rosmarinic acid. Additionally, 8-hydroxy salvigenin was detected in the nanoparticle of SGV (2.44 µg analyte/mg extract). It was thought that other phytochemical metabolites found in plants could not be detected because they were not released from the nanoparticles at a rate that could be analyzed by HPLC (Table 4; Figure 2).

**TABLE 2:** Phenolic metabolites detected in *S. libanotica* subsp. *linearis* extract.

Metabolites	Retention time	Area	Area (%)	µg analyte/mg extract
Quinic acid	6.326	205.60	1.516	0.22 ± 0.01
4-Hydroxybenzoic acid	19.336	37.02	0.254	0.30 ± 0.11
Chlorogenic acid	21.154	27.70	0.209	3.54 ± 0.89
Vanillic acid	21.896	100.23	0.678	0.24 ± 0.04
Caffeic acid	29.842	496.29	3.413	6.46 ± 2.26
2-Hydroxycinnamic acid	32.996	131.07	0.753	0.47 ± 0.10
Apigenin-7-O-neohesperidosid	33.753	176.25	1.289	1.69 ± 0.06
Rosmarinic acid	38.034	3,356.30	30.545	8.70 ± 2.51
*p*-Coumaric acid	39.901	959.37	6.909	4.42 ± 0.51
Unknown	35.542	1,533.201	11.289	Undetected
Unknown	36.138	559.383	4.119	Undetected
Unknown	37.745	2664.812	19.623	Undetected
Unknown	44.967	867.865	6.390	Undetected

**TABLE 3:** Phenolic metabolites detected in *S. germanicopolitana* subsp.*viridis* extract.

Metabolites	Retention time	Area	Area (%)	µg analyte/mg extract
Quinic acid	6.180	440.10	4.037	12.57 ± 3.88
Chlorogenic acid	23.506	149.21	1.164	4.35 ± 0.20
Caffeic acid	31.840	617.10	5.145	2.28 ± 0.26
Rosmarinic acid	32.540	362.86	3.871	1.97 ± 0.65
Luteolin	36.210	2930.59	17.5413	27.44 ± 3.69
*p*-Coumaric acid	37.810	1,245.36	8.262	20.03 ± 4.29
8-hydroxy salvigenin	40.119	490.90	2.896	2.74 ± 1.83
Unknown	35.712	4,393.288	29.999	Undetected
Unknown	45.091	805.232	5.496	Undetected

**TABLE 4:** Phenolic metabolites detected in nanoparticles.

Metabolites µg analyte/mg extract		
	SGV	SLL
Rosmarinic acid	1.79 ± 0.07	7.46 ± 0.47
8-hydroxy salvigenin	2.44 ± 0.16	

### 3.3 Antioxidant activity of different extracts and extract-loaded nanoparticle

The antioxidant activities of different extracts from the plant and extract-loaded nanoparticles were comparatively investigated by DPPH, FRAP and CUPRAC methods. It was determined that SGV (IC_50_: 0.028 mg/mL) and SLL (IC_50_:0.039 mg/mL) 70% ethanol extracts showed the highest DPPH radical scavenging activity compared to other extracts and hence these extract-loaded nanoparticles were prepared and characterized. A second comparison was made between the antioxidant activity of the crude extracts and the nanoparticles. The findings obtained showed that both 70% ethanol extractloaded nanoparticles showed lower DPPH radical scavenging activity than the crude extracts. Upon comparing plant species, it was shown that the nanoparticle loaded with SLL 70% ethanol extract (IC_50_: 3.154 mg/mL) has a greater capacity to scavenge free radicals. All plant extracts and extract-loaded nanoparticles demonstrated significantly lower capacity for radical scavenging when compared to ascorbic acid (IC_50_: 0.004 mg/mL), which was used as a reference for the potentials of radical scavenging (Table 1). The CUPRAC test results (Table 1) showed that SGV (2.538 mM troloxE/mg extract) and SLL (2.278 mM troloxE/mg extract) 70% ethanol extracts have a higher Cu(II) to Cu(I) reduction potential than other extracts. Crude extracts were shown to have higher CUPRAC values than nanoparticles when the CUPRAC values of 70% extract-loaded nanoparticles and crude extracts were evaluated. The findings showed that SLL 70% extract-loaded nanoparticles (0.030 mM troloxE/mg extract) showed higher copper (II) ion reduction than SGV 70% extract-loaded nanoparticles (0.022 mM troloxE/mg extract). Findings showed that the ascorbic acid (5.831 mM troloxE) had a higher Cu(II) to Cu(I) reduction potential than the 70% ethanol extracts and nanoparticles obtained from SGV and SLL. It was discovered that SGV (1.718 mMFeSO_4_/mg extract) and SLL (1.448 mMFeSO_4_/mg extract) 70% ethanol extracts had more iron reducing antioxidant power than other extracts. The extracted-loaded nanoparticles FRAP values were found to be lower than the raw extracts. The iron (III) ion reduction potential of the SLL extract-loaded nanoparticles (0.018 mMFeSO_4_/mg extract) was found to be greater than that of the SGV extract-loaded nanoparticles (0.013 mMFeSO_4_/mg extract). This study also found that the FRAP values of all SGV and SLL extracts and extract-loaded nanoparticles (Table 1) were lower than those of ascorbic acid (7.808 mMFeSO_4_).

### 3.4 Enzyme inhibition activity of different extracts and extract -loaded nanoparticles

The findings of the comparison of the potentials of the plant extracts and the extract-loaded nanoparticles inhibiting the enzymes acetylcholinesterase and urease are shown in Table 5. It was determined that SGV 70% ethanol extract (IC_50_: 0.099 mg/mL) and SLL 70% ethanol extract (IC_50_: 0.061 mg/mL) had higher urease enzyme inhibition potential than other extracts. The anti-urease activity of the nanoparticles loaded with SLL 70% ethanol extract (IC50: 7.21 mg/mL) was found to be greater than that of the nanoparticles made with SGV 70% ethanol extract (IC50: 9.501 mg/mL). Crude extracts demonstrated more enzyme inhibition when the potentials of both nanoparticles and crude extracts on the urease enzyme were assessed. It was also observed that unloaded nanoparticles (IC_50_: 12.753 mg/mL) showed a certain level of enzyme inhibition and that the activity increased significantly when the extract was loaded. These findings suggested that the polymer (SA) used in nanoparticle preparation have also biological activity potential.

**TABLE 5:** The ability of different extracts and extract-loaded nanoparticles to inhibit enzymes.

Exctracts/Ass ays			SGV					SLL				Standards
	Petroleum ether	Chloroform	70% ethanol	70% ethanol loaded nanoparticle	Extract unloadedna noparticle	Petroleu m ether	Chloro form	70%ethanol	70% ethanol loadednanoparti cle	Extractunlo aded nanoparticl e	Thiourea	Galantamine
Urease inhibition (IC_50_:mg/mL)	0.195 ± 0.019*	0.152 ± 0.135*	0.099 ± 0.028*	9.501 ± 0.054*	12.753 ± 1.674*	0.076 ± 0.002*	0.089 ± 0.033*	0.061 ± 0.024*	7.21 ± 2.940*	12.753 ± 1.674*	0.002 ± 0.001	
Acetylcholine steraseinhibiti on (%) (200 μg/mL)	68.53 ± 0.313*	74.623 ± 1.231*	77.137 ± 0.209*	23.508 ± 0.974*	12.441 ± 1.921*	72.574 ± 0.552*	74.262 ± 1.934*	77.895 ± 2.455*	42.726 ± 7.253*	12.441 ± 1.921*		85.289 ± 0.06

Thiourea positive control for Urease assays; Galantamine positive control for anticholinesterase assays. Values are mean of triplicate determination (n = 3) ± standard deviation; *P < 0.05 compared with the positive control; SGV: *S. germanicopolitana* subsp. *viridis;* SLL*: S. libanotica* subsp. *linearis*.

Furthermore, an analysis of the acetylcholinesterase enzyme inhibition capacity of various extracts from both plants revealed that the 70% ethanol extracts (SGV:77.137%; SLL: 77.895%) of each plant species had a greater potential for enzyme inhibition than the other extracts. The anticholinesterase activity of the nanoparticles loaded with SLL 70% extract (42.726%) was found to be higher than that of the nanoparticles loaded with SGV 70% ethanol extract (23.508%). The acetylcholinesterase enzyme inhibition potential of the nanoparticles made from both plant species was found to be lower than that of the raw extracts. The presence of a certain degree of enzyme inhibition in unloaded nanoparticles (12.441%) indicated that the polymer utilized in the nanoparticle production process (SA) has biological activity (Table 5). The results of this investigation indicated that these formulations could be employed as anti-urease and anticholinesterase medicines in the future, given the benefits of utilizing nanoparticles in the treatment. Table 5.

### 3.5 Characterization of the nanoparticles

**FIGURE 3:**
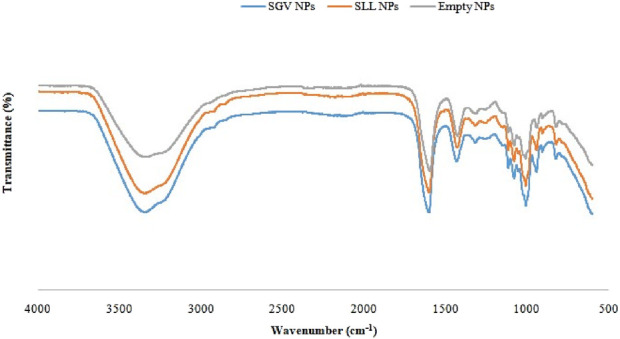
FTIR spectra of SGV NPs, SLL NPs and unloaded NPs.

Fourier-transform infrared (FTIR) spectroscopy was used to examine the possible interactions between encapsulated SGV/SLL and the alginate matrix of the nanoparticles. Characteristic peaks of the SA matrix are seen at around 3,300 cm^−1^, 1,600 cm^−1^, 1,400 cm^−1^ and 1,000 cm^−1^ in the Figure 3. There are broad bands between 3,000 cm^−1^ and 3,500 cm^−1^ in the spectra of all the nanoparticle samples which shows the O-H bonds of the hydroxyl groups. There are sharp peaks at around 1,600 cm^−1^ representing the asymmetric C=O bonds of the COO^¯^ groups. There are also peaks at around 1,400 cm^−1^ representing symmetric bonds of the COO^¯^ groups. There are sharp peaks at around 1,000 cm^−1^ which are attributed to single bonds of the C-O-C stretching vibrations (De Silva et al., 2024; Çalışkan Salihi et al., 2021; Demirhan et al., 2021; Wang et al., 2017). There are no changes were observed in the peak positions after loading of the SGV and SLL which shows the loading of the extracts to the alginate nanoparticles occurred through physical interactions and the extracts were physically dispersed across the alginate chains (Severino et al., 2019).

**FIGURE 4:**
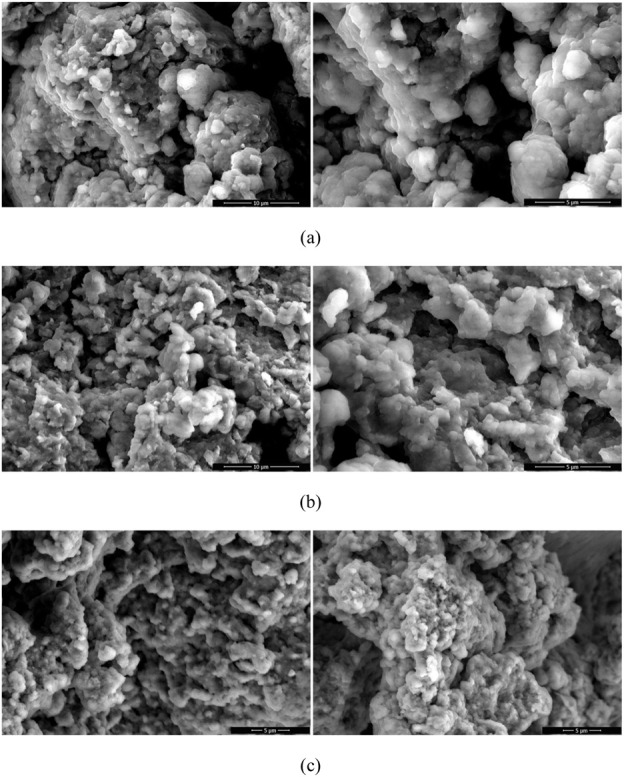
SEM images of SGV NPs: **(A)**, SLL NPs **(B)** and empty NPs **(C)**.

Scanning electron microscopy (SEM) was used to investigate the surface morphology and the sizes of the nanoparticle formulations. SEM images of SGV NPs, SLL NPs and empty NPs were given in Figure 4. The sizes of the samples prepared are at the nanoscale and they have the spherical heterogenous shapes with smooth surfaces which are visibly seen in the SEM images (Anirudhan et al., 2017).

**FIGURE 5:**
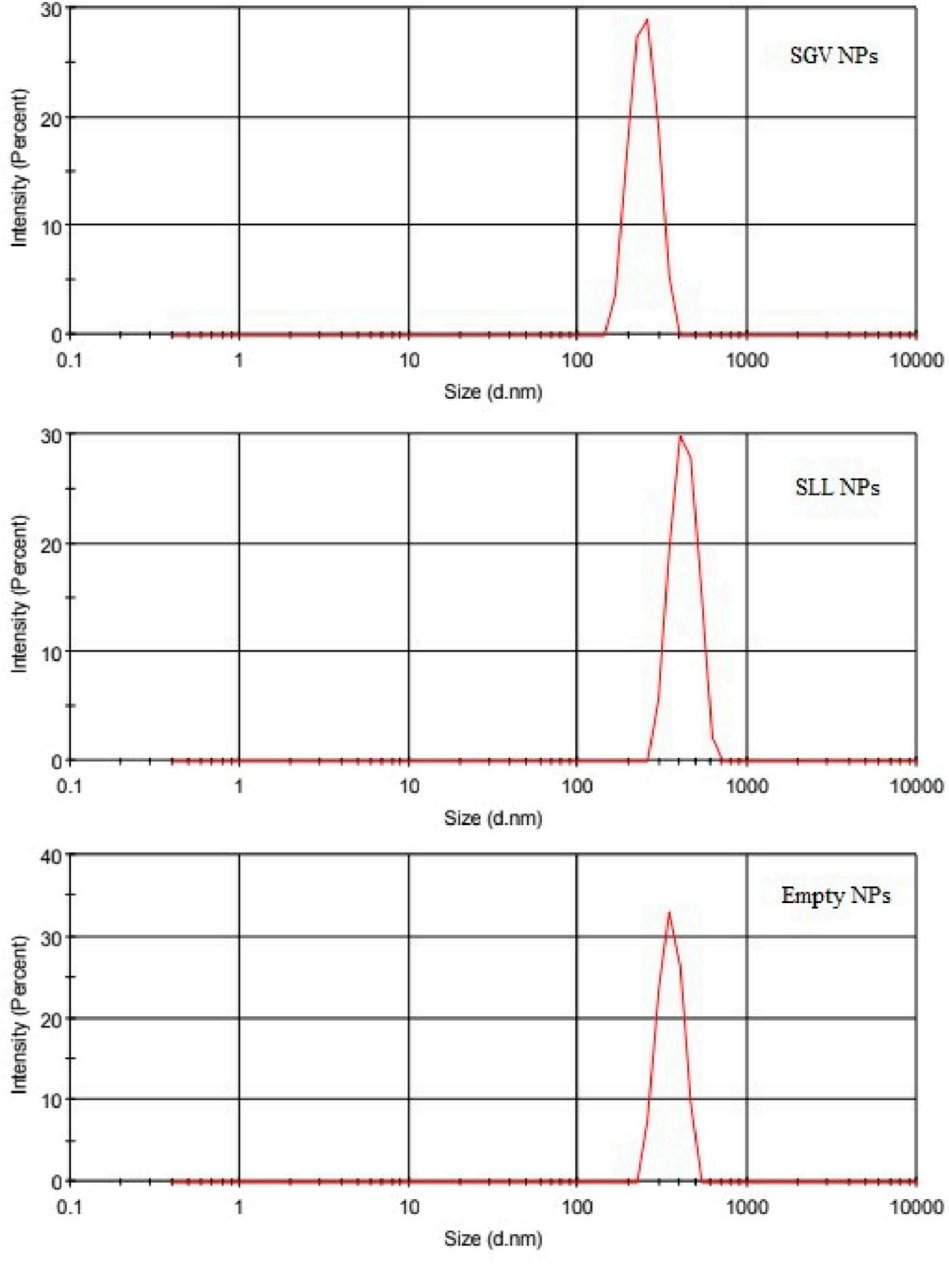
Intensity-based particle size distribution of SGV NPs: SLL NPs and empty NPs.

The particle size analysis of the nanoparticles by dynamic light scattering (DLS) technique are shown in Figure 5 as intensity (%) based particle size distribution versus size in nanometers. Results of hydrodynamic size and polydispersity index (PdI) of the nanoparticles are given in Table 6. PdI values indicates the uniformity of the sizes of the nanoparticles in dispersions. According to the results (Figure 5; Table 6), SGV NPs, SLL NPs and empty NPs have average diameters of 487.1, 582.8, 685.8 nm; sizes at peak maximum 244.2, 422.2, 350.3 nm and PdI values of 0.471, 0.407, 0.660. Figure 5 also shows that there is no aggregates or large particles in the dispersion system. The Z-average values and the size at peak maximum showed that the particles are at the nanoscale with reasonable polidispersity index (PdI) values (Soltanzadeh et al., 2021).

**TABLE 6:** Average diameter, size at peak maximum and poly-dispersity index (PdI), encapsulation efficiency (EE) and loading capacity (LC) of SGV NPs, SLL NPs and empty NPs.

	Z-Average (d.nm)	Size (d.nm)	PdI	Encapsulation efficiency, EE (%)	Loading capacity, LC (%)
SGV NPs	357.0	282.4	0.429	17.66	3.45
SLL NPs	582.8	422.2	0.407	14.98	3.35
Empty NPs	685.8	350.3	0.660	-	-

Encapsulation of the SGV and SLL extracts shifted the average diameters of the nanoparticles to smaller particle sizes with lower PdI values indicating improved uniformity of the particle size distribution, in comparison with the empty NPs. This result expresses that the presence of the phenolic rich extracts showed surface activity and improved the emulsifying properties of the NPs (Flamminii et al., 2020).

The encapsulation efficiency (EE) and the loading capacity (LC) of the nanoparticles were calculated using the Equation 1 and Equation 2, respectively. EE was found to be 17.66% for SGV NPs and 14.98% for SLL NPs while LC was found to be 3.45% for SGV NPs and 3.35% for SLL NPs as given in Table 6. SLL and SGV were shown similar efficiency and capacity for the encapsulation with sodium alginate matrix and the calculated results showed the encapsulation limitations of these extracts across alginate chains (Soltanzadeh et al., 2021).

**FIGURE 6:**
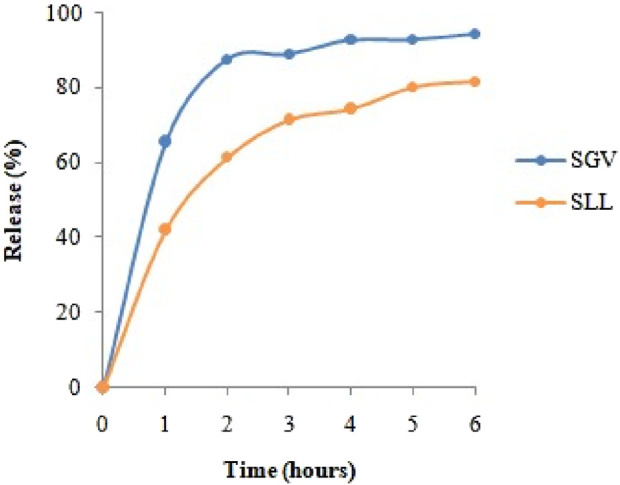
*In vitro* release profiles of encapsulated SGV and SLL.

*In-vitro* release abilities of the SGV NPs and SLL NPs were studied in PBS medium to show their capability to be used in drug delivery applications. Release profiles of SGV NPs and SLL NPs were shown in Figure 6. There is a burst release effect in the first hour for the both delivery systems which was followed by a gradual release in the following hours. About 90% of the SGV was released gradually from the SGV NPs in the next 5 h while about 80% of the SLL was released gradually from the SLL NPs in the next 5 h (Ji et al., 2019). The release profile of the both encapsulated nanoparticles showed that the formulations produced are promising natural drug delivery systems synthesized using the green chemistry approach.

## 4 Discussion

Phenolic metabolites were shown to have potent antioxidant, antimicrobial, anticarcinogenic, antiinflammatory, anti-urease and anticholinesterase properties in the literature review. Thus far, over 8,000 phenolic metabolites have been found in naturally occurring sources (Sotirios et al., 2020; Yuanyuan et al., 2022). In *Sideritis* species (*S. scardica* and *S. raeseri*), 5,7-hydroxyflavones (apigenin and luteolin) and 8-hydroxyflavones (hypolaetin (8-OH luteolin)) and their derivatives are present as phenolic substances. Later researches have verified the existence of 8-hydroxyflavones such isoscutellarein, hypolaetin, and their methoxy derivatives, which are extremely distinctive for *Sideritis* species. Additionally, the following hydroxycinnamic acid derivatives were found in *Sideritis* extracts: *p*-coumaric acid, 3-*O-*caffeoylquinic acid, and 5-*O-*caffeoylquinic acid (the two main representatives of chlorogenic acids) feruloylquinic acid and 4-*O*-glucoside. The other research verified that the chemical makeup of plants from the genus *Sideritis* (samples of *S. scardica* and *S. raeseri*) gathered from the Balkan countries is identical. The phenolic metabolites contained in *Sideritis* species provide strong biological effects to these species (Petreska et al., 2011; Ibraliu et al., 2015; Stanoeva et al., 2015; Dorota et al., 2020).

The Soxhlet technique was used to create a methanol extract from the aerial parts of the *S. libanotica* subsp. *linearis* (SLL*)*, and the IC_50_ value for its ability to scavenge DPPH radicals was found to be 109 μg/mL. It was discovered that it demonstrated less radical scavenging activity than the reference standards, ascorbic acid (3.80 μg/mL) and BHT (18.00 μg/mL) (Tepe et al., 2006). In another study, the analysis of total phenolic metabolites in 80% methanol extract of SLL plant was investigated and it was found that the extract contained 10.33 ± 0.45 g GAE/kg dw phenolic extract.

The value of the DPPH radical scavenging activity is 14.08 ± 0.86 g dw/g DPPH in appearance. The primary metabolites in this extract were identified as *p*-coumaric, caffeic, ferulic acids, quercetin, morin, and apigenin (Dincer et al., 2017). Furthermore, 3′-*O*-methylhypolaetin 7-*O*-[6″- *O*-acetyl-βD-allopyranosyl-(1-2)]-6″-*O*-acetyl-β-D-glucopyranoside and sideridiol, which have been found to have potent antioxidant activity, have been isolated from the methanol extract of this plant (Demirtas et al., 2011). Investigations on the cytotoxic effects of the SLL plant’s methanol extract on Vero, HeLa, and C6 cell lines revealed that it was effective at cytotoxic activities at a concentration of 250 μg/mL (Demirtas et al., 2009). It was discovered that *S. aureus* was resistant to the methanol extract of the SLL plant (MIC: 64 μg/mL). Additionally, a strong scavenging action of the DPPH radical (IC50: 0.0133 mg/mL) was discovered (Güven et al., 2021).

In this study, the antioxidant activity of different extracts from the *Sideritis libanotica* subsp. *linearis* species was examined. It was determined that the 70% ethanol extract (IC_50_:39 μg/mL) of the plant showed significant DPPH radical scavenging activity. Compared to the above study, this study determined that 70% ethanol extract had higher radical scavenging activity potential. It is thought that the difference between the studies is due to agroecological conditions, the time of collection and the solvent used in the extraction process. In addition, in this study, the phenolic metabolites contained in 70% ethanol extract of the plant were analysed in amounts similar to the above studies. The chemicals from *Sideritis* species that were examined in this study yielded findings that were consistent with those of other investigations. In the literature review of the *Sideritis germanicopolitana* subsp. *viridis* (SGV), no detailed study on the chemical composition and biological activities of this species was found. The biological activity and chemical composition of various extracts and bioactive extract-loaded nanoparticles of these two *Sideritis* species were examined in depth for the first time in this study. When the biological activities of 70% ethanol extracts from both *Sideritis* species and nanoparticles loaded with these extracts were compared, it was determined that the extracts had lower activity than the nanoparticles. The actual amount of the extracts calculated by using Loading Capacity (%) values has been considered for the comparison which are 3.45% for SGV and 3.35% for SLL. As in the study, when the biological activities of plant extract-loaded nanoparticles and free forms of the same extracts are compared, there are studies in the literature that the free forms are more active biologically. In our study, the crude plant extract also showed higher activity than the produced nanoformulations. Since crude plant extracts may show toxicity, their direct use in pharmaceutical applications is limited. To solve this problem, plant extracts must be loaded into a carrier system. The advantages of carrier systems are that, in addition to reducing toxicity, they provide controlled release and increase the bioavailability of the active ingredients. As a result, even though the produced nanoparticle showed lower activity than the extract, produced formulation has enhanced its potential to be applied in the treatment. As in our study, when the biological activities of plant extract-loaded nanoparticles and free forms of the same extracts are compared, there are studies in the literature that the free forms are more biologically active. Eventually, in order to be used in real life applicatons, they must be produced into a suitable and safe formulation (Mishra et al., 2021; Ge et al., 2022; Maqsoudlou et al., 2022; Li et al., 2021; Sinlapapanya et al., 2024).

## 5 Conclusion

Bioactive extracts of SLL and SGV were encapsulated into natural polymer-based nanoparticles using the green chemistry approach without using any additives or surface-active agents following an environmentally friendly route, in this study. Physicochemical characterization of the nanoparticles was done besides the *in vitro* release study of the active agents which show the potential of these formulations as drug delivery systems for the controlled release and diminished side effects. The findings of this study showed that the 70% ethanol extracts from SLL and SGV had the highest antiurease, anticholinesterase, and antioxidant properties. Along with producing and characterizing nanoparticles from these extracts, a qualitative and quantitative analysis of the phytochemical contents of these extracts was conducted. It was also analyzed that luteolin and *p*-coumaric acid was observed in high content in the SGV extract while rosmarinic acid, caffeic acid and *p*-coumaric acid was found in high concentration in SLL extract. Crude extracts and nanoparticles loaded with extracts from both *Sideritis* species were compared for their antioxidant, antiurease, and anticholinesterase properties. The data demonstrated that the crude extracts had higher biological activity potential than the nanoparticles. The nanoparticle formulations developed as a result of this work have significant biological activities, it is thought that these formulations can be used as antioxidants, antiurease and anticholinesterase agents in the future after their cytotoxic effects and *in vivo* experiments are performed.

## Data Availability

The raw data supporting the conclusions of this article will be made available by the authors, without undue reservation.

## References

[B1] Ahmad R. Srivastava R. Ghosh S. Khare S. K. (2021). Phytochemical delivery through nanocarriers: a review. Colloids Surf. B Biointerfaces. 197, 111389. 10.1016/j.colsurfb.2020.111389 33075659

[B2] Anand P. Singh B. (2013). A review on cholinesterase inhibitors for Alzheimer’s disease. Arch. Pharm. Res. 36 (4), 375–399. 10.1007/s12272-013-0036-3 23435942

[B3] Anirudhan T. S. Anila M. M. Franklin S. (2017). Synthesis characterization and biological evaluation of alginate nanoparticle for the targeted delivery of curcumin. Mater Sci. Eng. C 78, 1125–1134. 10.1016/j.msec.2017.04.116 28575948

[B4] Apak R. Güçlü K. Özyürek M. Karademir S. E. (2004). Novel total antioxidant capacity index for dietary polyphenols and vitamins c and e, using their cupric ion reducing capability in the presence of neocuproine: CUPRAC method. J. Agric. Food Chem. 52 (26), 7970–7981. 10.1021/jf048741x 15612784

[B5] Atas M. Eruygur N. Sozmen F. Ergul M. Ergul M. Akpulat H. A. (2019). Evaluation of various biological activities of endemic *Sideritislibanotica* Extracts. Not. Sci. Biol. 11 (2), 210–217. 10.15835/nsb11210442

[B6] Azizian H. Nabati F. Sharifi A. Siavoshi F. Mahdavi M. Amanlou M. (2012). Large-scale virtual screening for the identification of new *Helicobacter pylori* urease inhibitor scaffolds. J. Mol. Model. 18 (7), 2917–2927. 10.1007/s00894-011-1310-2 22139480

[B7] Bakadia B. M. Boni B. O. O. Ahmed A. A. Q. Yang G. (2021). The impact of oxidative stress damage induced by the environmental stressors on COVID-19. Life Sci. 264, 118653. 10.1016/j.lfs.2020.118653 33115606 PMC7586125

[B8] Barja G. (2004). Free radicals and aging. Trends Neurosci. 27 (10), 595–600. 10.1016/j.tins.2004.07.005 15374670

[B9] Bayan Y. Aksit H. (2016). Antifungal activity of essential oils and plant extracts from *Sideritisgermanicopolitana* BORNM. Growin in Turkey. EJBPC. 26 (2), 333–337.

[B10] Benzie I. F. Strain J. J. (1996). The ferric reducing ability of plasma (FRAP) as a measure of “antioxidant power”: the FRAP assay. Anal. Biochem. 239, 70–76. 10.1006/abio.1996.0292 8660627

[B11] Çalışkan Salihi E. Wang J. Kabacaoğlu G. Kırkulak S. Šiller L. (2021). Graphene oxide as a new generation adsorbent for the removal of antibiotics from waters. Sep. Sci. Technol. 56 (3), 453–461. 10.1080/01496395.2020.1717533

[B55] Çalışkan Salihi E. Zarrabi A. Zarepour A. Gürboğa M. Hasan Niari Niar S. Özakpınar Ö. B. (2025). Ambient pressure dried graphene oxide-silica composite aerogels as pharmaceutical nanocarriers. Journal of Sol-Gel Science and Technology 113 (2), 548–558. 10.1007/s10971-024-06624-1

[B12] De Silva N. D. Attanayake A. P. Karunaratne D. N. Arawwawala L. D. A. M. Pamunuwa G. K. (2024). Synthesis and bioactivity assessment of Coccinia grandis L. extract encapsulated alginate nanoparticles as an antidiabetic drug lead. J. Microencapsul. 41 (1), 1–17. 10.1080/02652048.2023.2282964 37966469

[B13] Demirhan K. BingolOzakpinar O. Çalışkan Salihi E. (2021). Green and one step modification of graphene oxide using natural substances. Fuller. nanotub. Carbon Nanostructures 29 (9), 716–723. 10.1080/1536383x.2021.1884074

[B14] Demirtas I. Sahin A. Ayhan B. Tekin S. Telci I. (2009). Antiproliferative effects of the methanolic extracts of *Sideritis libanotica* Labill. subsp. linearis. Rec. Nat. Prod. 3 (2), 104–109.

[B15] Demirtas I. Ayhan B. Sahin A. Aksit H. Elmastas M. Telci I. (2011). Antioxidant activity and chemical composition of *Sideritis libanotica* Labill. Ssp. linearis (Bentham) Borm. Lamiaceae. Nat.Prod. Res. 25 (16), 1512–1523. 10.1080/14786410903293191 20544498

[B16] Dincer C. Torun M. Tontul I. Topuz A. Sahin-Nadeem H. Gokturk R. S. (2017). Phenolic composition and antioxidant activity of *Sideritis lycia* and *Sideritis libanotica* subsp. *linearis*: effects of cultivation, year and storage. J. Appl. Res. Med. Aromat. Plants 5, 26–32. 10.1016/j.jarmap.2016.09.006

[B17] Dorota Z. Kamila K.-W. Joanna O. Kacper Z. (2020). Polyphenols and other bioactive compounds of *Sideritis*plants and their potential biological activity. Molecules 25, 3763–3780. 10.3390/molecules25163763 32824863 PMC7464829

[B18] Ellman G. L. Courtney K. D. Andres Jr V. Featherstone R. M. (1961). A New and rapid colorimetric determination of acetylcholinesterase activity. Biochem. Pharmacol. 7 (2), 88–95. 10.1016/0006-2952(61)90145-9 13726518

[B19] Fang Y. Z. Yang S. Wu G. (2002). Free radicals, antioxidants, and nutrition. Nutrition 18 (10), 872–879. 10.1016/s0899-9007(02)00916-4 12361782

[B20] Fisher A. Hanin I. Lachman C. (2012). Alzheimer’s and Parkinson’s diseases: strategies for research and development (Springer Science and Business Media), 29.

[B21] Flamminii F. Di Mattia C. D. Nardella M. Chiarini M. Valbonetti L. Neri L. (2020). Structuring alginate beads with different biopolymers for the development of functional ingredients loaded with olive leaves phenolic extract. Food Hydrocoll. 108, 105849. 10.1016/j.foodhyd.2020.105849

[B56] Formisano C. Oliviero F. Rigano D. Arnold N. A. Senator F. (2015). Comparative chemical composition and antioxidant properties of the essential oils of three sideritis libanotica subspecies. Nat. Prod. Commun. 10 (6), 1075–1078. 10.1177/1934578X1501000670 26197555

[B22] Ge X. Cao Z. Chu L. (2022). The Antioxidant effect of the metal and metal-oxide nanoparticles. Antioxidants 11 (2022), 791–797. 10.3390/antiox11040791 35453476 PMC9030860

[B23] Ghous T. Akhtar K. Nasim F. U. H. Choudhry M. A. (2010). Screening of selected medicinal plants for urease inhibitory activity. Biol. Med. 2, 64–69.

[B24] González-Burgos E. Carretero M. E. Gómez-Serranillos M. P. (2011). *Sideritis* spp.: uses, chemical composition and pharmacological activities-A review. J. Ethnopharmacol. 135 (2), 209–225. 10.1016/j.jep.2011.03.014 21420484

[B25] Graham D. Y. Miftahussurur M. (2018). *Helicobacter pylori* urease for diagnosis of *Helicobacter pylori* infection: a mini review. J. Adv. Res. 13, 51–57. 10.1016/j.jare.2018.01.006 30094082 PMC6077137

[B26] Gülsoy Toplan G. Kürkçüoğlu M. Göger F. Taşkın T. Civaş A. İşcan G. (2022). Phytochemical screening and biological evaluation of Salvia hydrangea DC. ex Benth. growing in eastern Anatolia. S Afr. J. Bot. 147, 799–807. 10.1016/j.sajb.2022.03.021

[B27] Güven U. M. Kayiran S. D. Aygül A. Nenni M. Kirici S. (2021). Design of microemulsion formulations loaded *Scutellariasalviifolia* Benth, *Sideritislibanotica* Labill. subsp. *linearis* (Bentham) Bornm, and *Ziziphoraclinopodioides* Lam. extracts from Turkey and *in vitro* evaluation of their biological activities. Turk. J. Bot. 45, 789–799. 10.3906/bot-2108-50

[B28] Hashem H. M. Motawea A. Kamel A. H. Bary E. A. Hassan S. S. (2022). Fabrication and characterization of electrospun nanofibers using biocompatible polymers for the sustained release of venlafaxine. Sci. Rep. 12 (1), 18037. 10.1038/s41598-022-22878-7 36302929 PMC9614003

[B29] Hcini K. Lozano-Pérez A. A. Luis Cenis J. Quílez M. José Jordán M. (2021). Extraction and encapsulation of phenolic compounds of tunisian rosemary (Rosmarinus officinalis L.) extracts in silk fibroin nanoparticles. Plants 10 (11), 2312. 10.3390/plants10112312 34834676 PMC8618009

[B30] Holst B. Williamson G. (2008). Nutrients and phytochemicals: from bioavailability to bioefficacy beyond antioxidants. CRBIOT 19 (2008), 73–82. 10.1016/j.copbio.2008.03.003 18406129

[B31] Ibraliu A. Trendafilova A. B. Anđelković B. D. Qazimi B. Gođevac D. M. Shengjergji D. (2015). Comparative study of balkan *sideriti*s species from Albania, Bulgaria and Macedonia. Eur. J. Med. Plants. 5, 328–340. 10.9734/ejmp/2015/14389

[B32] Jahangirian H. Lemraski E. G. Webster T. J. Rafiee-Moghaddam R. Abdollahi Y. (2017). A review of drug delivery systems based on nanotechnology and green chemistry: green nanomedicine. Int. J. Nanomedicine 12, 2957–2978. 10.2147/IJN.S127683 28442906 PMC5396976

[B33] Ji M. Sun X. Guo X. Zhu W. Wu J. Chen L. (2019). Green synthesis, characterization and *in vitro* release of cinnamaldehyde/sodium alginate/chitosan nanoparticles. Food Hydrocoll. 90, 515–522. 10.1016/j.foodhyd.2018.12.027

[B34] Kan Y. Kan A. Ayran İ. Çelik S. A. (2018). Essential oil yield and compositions of endemic mountain tea (*Sideritislibanotica*Labill. ssp. *linearis* (Bentham) Borm. and *Sideritisbilgerana* PH Davis) cultivated in Konya ecological conditions of Turkey. Int. J. Agric. Environ. Food Sci. 1, 204–205. 10.31015/jaefs.18038

[B35] Maqsoudlou A. Assadpour E. Mohebodini H. Jafari S. M. (2022). The influence of nanodelivery systems on the antioxidant activity of natural bioactive compounds. Crit. Rev. Food Sci. 10.1080/10408398.2020.1863907 33356489

[B36] Martinez-Ballesta M. Gil-Izquierdo A. Garcia-Viguera C. Dominguez-Perles R. (2018). Nanoparticles and controlled delivery for bioactive compounds: outlining challenges for new “Smartfoods”for health. Foods. 7, 72–76. 10.3390/foods7050072 29735897 PMC5977092

[B37] Martinez-Perez B. Quintanar-Guerrero D. Tapia-Tapia M. Cisneros-Tamayo R. ZambranoZaragoza M. L. Alcala-Alcala S. (2018). Controlled-release biodegradable nanoparticles: from preparation to vaginal applications. Eur. J. Pharm. Sci. 115 (2018), 185–195. 10.1016/j.ejps.2017.11.029 29208486

[B38] Mishra P. Kumar A. Panda G. (2019). Anti-cholinesterase hybrids as multi-target-directed ligands against Alzheimer’s disease (1998-2018). Bioorg. Med. Chem. 27 (6), 895–930. 10.1016/j.bmc.2019.01.025 30744931

[B39] Mishra S. Sahani S. Pal V. (2021). Encapsulation of herbal extracts. Sustain. Agric. Rev. 55 Micro Nano Eng. Food Sci. 1, 115–133. 10.1007/978-3-030-76813-3_5

[B40] Özkan G. Krüger H. Schulz H. Özcan M. (2005). Essential oil composition of three *Sideritis* species used as herbal teas in Turkey. J. Essent. Oil-Bear. Plants 8 (2), 173–177. 10.1080/0972060x.2005.10643439

[B41] Petreska J. Stefova M. Ferreres F. Moreno D. A. Tomás-Barberán F. A. Stefkov G. (2011). Potential bioactive phenolics of Macedonian *Sideritis*species used for medicinal “Mountain Tea”. Food Chem. 125, 13–20. 10.1016/j.foodchem.2010.08.019

[B42] Rajaonarivony M. Vauthier C. Couarraze G. Puisieux F. Couvreur P. (1993). Development of a new drug carrier made from alginate. J. Pharm. Sci. 82 (9), 912–917. 10.1002/jps.2600820909 8229689

[B43] Severino P. da Silva C. F. Andrade L. N. de Lima Oliveira D. Campos J. Souto E. B. (2019). Alginate nanoparticles for drug delivery and targeting. Curr. Pharm. Des. 25 (11), 1312–1334. 10.2174/1381612825666190425163424 31465282

[B44] Sevindik E. Gübeş İ. Murathan Z. T. Tümen G. (2021). Determination of total phenolic content, total flavonoid content and total antioxidant capacity in some endemic *Sideritis*L. (Lamiaceae) species grown in Turkey. Eur. J. Biol. 11 (2), 260–266. 10.5281/zenodo.4660074

[B45] Sinlapapanya P. Buatong J. Palamae S. Nazeer R. A. Zhang B. Prodpran T. (2024). Ethanolic cashew leaf extract encapsulated in tripolyphosphate–chitosan complexes: characterization, antimicrobial, and antioxidant activities. Colloids Interfaces 8 (2024), 52–56. 10.3390/colloids8050052

[B46] Soltanzadeh M. Peighambardoust S. H. Ghanbarzadeh B. Mohammadi M. Lorenzo J. M. (2021). Chitosan nanoparticles as a promising nanomaterial for encapsulation of pomegranate (*Punica granatum* L.) peel extract as a natural source of antioxidants. Nanomaterials 11 (6), 1439. 10.3390/nano11061439 34072520 PMC8228277

[B57] Sotirios K. Charalampos P. Vassiliki O. (2020). Phenolic acids of plant origin—a review on their antioxidant activity *in vitro* (o/w emulsion systems) along with their *in vivo* health biochemical properties. Foods 9 (4), 534–537. 10.3390/foods9040534 32344540 PMC7231038

[B47] Stanoeva J. P. Stefova M. Stefkov G. Kulevanova S. Alipieva K. Bankova V. (2015). Chemotaxonomic contribution to the *Sideritis* species dilemma on the Balkans. Biochem. Syst. Ecol. 61, 477–487. 10.1016/j.bse.2015.07.008

[B48] Stingl K. De Reuse H. (2005). Staying alive overdosed: how does *Helicobacter pylori* control urease activity? Int. J. Med. Microbiol. 295 (5), 307–315. 10.1016/j.ijmm.2005.06.006 16173497

[B49] Taşkın D. Yılmaz B. N. Taşkın T. Omurtag G. Z. (2021). The influence of different extraction methods/solvents on composition, biological activities and ADMET predictions of phenolics in Tribulus terrestris. Braz. Arch. Biol. Technol. 64, e21210249. 10.1590/1678-4324-2021210249

[B50] Tepe B. Sokmen M. Akpulat H. A. Yumrutas O. Sokmen A. (2006). Screening of antioxidative properties of the methanolic extracts of *Pelargonium endlicherianum*Fenzl. Verbascumwiedemannianum Fisch. &Mey., SideritislibanoticaLabill. Subsp. linearis(Bentham). 10.1016/j.foodchem.2005.05.046

[B51] Valko M. Leibfritz D. Moncol J. Cronin M. T. Mazur M. Telser J. (2007). Free radicals and antioxidants in normal physiological functions and human disease. Int. J.Biochem. Cell. Biol. 39 (1), 44–84. 10.1016/j.biocel.2006.07.001 16978905

[B52] Wang J. Çalışkan Salihi E. Šiller L. (2017). Green reduction of graphene oxide using alanine. Mater. Sci. Eng. C 72, 1–6. 10.1016/j.msec.2016.11.017 28024564

[B53] Wei F. Jinglou C. Yaling C. Yongfang L. Liming C. Lei P. (2010). Antioxidant, free radical scavenging, anti-inflammatory and hepatoprotective potential of the extract from *Parathelypterisnipponica* (Franch.et Sav.) Ching. J. Ethnopharmacol. 130, 521–528. 10.1016/j.jep.2010.05.039 20669367

[B58] Yuanyuan Z. Ping C. Guanghui C. Guanghui Z. (2022). A brief review of phenolic compounds identified from plants: their extraction, analysis, and biological activity. Nat. Prod. Commun. 17 (1), 1–8. 10.1177/1934578X211069721

[B54] Zeng X. Jiang W. Du Z. Kokini J. L. (2023). Encapsulation of tannins and tannin-rich plant extracts by complex coacervation to improve their physicochemical properties and biological activities: a review. Crit. Rev. Food Sci. Nutr. 63 (18), 3005–3018. 10.1080/10408398.2022.2075313 35549567

